# Advances in Optical Fiber Sensors for Multi-Analyte Biochemical Detection

**DOI:** 10.3390/bios16070367

**Published:** 2026-07-06

**Authors:** Jianwei Huang, Fan Jia, Shaoxiang Duan, Bo Liu

**Affiliations:** Institute of Modern Optics, Tianjin Key Laboratory of Optoelectronic Sensor and Sensing Network Technology, Nankai University, Tianjin 300350, China; 1120240247@mail.nankai.edu.cn (J.H.); fanjia@nankai.edu.cn (F.J.); liubo@nankai.edu.cn (B.L.)

**Keywords:** optical fiber biochemical sensor, multi-analyte detection, biochemical sensing, multiplexed sensing, multi-channel, surface plasmon resonance, optical fiber sensor arrays

## Abstract

Optical fiber multi-analyte biosensors have become an important cutting-edge technology for the simultaneous detection of multiple biochemical substances in complex samples due to their unique advantages such as small size, anti-interference capability, and remote and label-free detection. This paper systematically reviews the recent research progress of optical fiber multi-analyte biosensors in the field of simultaneous detection of various types of targets. The review is organized by detection target type and elaborates on the simultaneous detection of biomarkers and proteins, viruses and bacteria, biological metabolites and nutrients, heavy metal ions, gases, organic pollutants, cells, and mixed detection of different types of biochemical substances. The advantages and disadvantages of existing optical fiber multi-analyte biosensors are summarized. Key technical challenges are also discussed, including issues of selectivity, long-term stability, real-sample validation, and system integration that currently hinder practical deployment. Finally, the future challenges and development directions of optical fiber multi-analyte biosensors are briefly discussed, providing references for relevant research teams.

## 1. Introduction

Nowadays, with increasing attention to precision medicine, environmental monitoring, food safety, and public health, the demand for rapid, sensitive, and simultaneous detection of multiple biochemical substances in complex samples is growing swiftly [1,2]. This demand is particularly acute in specific diagnostic contexts: early cancer detection requires profiling of multiple circulating tumor biomarkers from a single blood draw; cardiovascular risk assessment benefits from simultaneous measurement of multiple protein markers; disease diagnosis increasingly relies on biomarker panels rather than single analytes; and infectious disease serology often needs to distinguish between different pathogens or antibody isotypes. Traditional biochemical analysis methods, such as spectral analysis [3], electrochemical analysis [4], enzyme-linked immunosorbent assay [5], polymerase chain reaction [6], mass spectrometry, and imaging analysis [7,8], although possessing high sensitivity and specificity, usually suffer from limitations such as cumbersome operation, long time consumption, high cost, and the requirement for professional personnel and large-scale equipment. More importantly, while techniques such as mass spectrometry, multiplex PCR, imaging cytometry, and bead-based immunoassays can achieve highly multiplexed detection, they remain constrained by instrument complexity, high cost, long turnaround times, and limited field deployability, which makes them less suitable for real-time and in situ applications. In clinical diagnosis, a single biomarker is usually limited by insufficient sensitivity and specificity, while the combined detection of multiple related biomarkers can significantly improve the accuracy of early disease diagnosis. Similarly, the simultaneous monitoring of multiple heavy metal ions or mixed gases in environmental assessment is crucial for effectively evaluating pollution levels and ecological risks. Therefore, the development of high-performance sensing technologies capable of parallel detection of multiple analytes has become an important frontier in the field of biochemical analysis.

Optical fiber biochemical sensors have developed vigorously over the past few decades due to their inherent advantages of small size, light weight, anti-electromagnetic interference, corrosion resistance, remote and distributed sensing, and good biocompatibility [9,10,11]. In particular, optical fiber sensors can convert biochemical recognition events into measurable optical signal changes through various optical principles, such as surface plasmon resonance [12], fluorescence [13], interference [14], grating [15], and evanescent wave [16], providing an ideal platform for highly sensitive and highly specific biochemical detection. It should be noted that while label-free detection is a valuable capability of optical fiber sensors, it is not exclusive to this technology, as other platforms such as quartz crystal microbalance and electrochemical sensors also offer label-free sensing. Nonetheless, the combination of label-free detection with the unique optical properties of fibers makes them particularly attractive for a wide range of biochemical applications. They have been applied to the detection of various targets, including biomarkers [17], proteins [18], viruses [19], bacteria [20], metabolites [21], heavy metal ions [22], gases [23], organic pollutants [24], and even cells [25]. However, most early optical fiber sensors were designed for a single target analyte [26,27], making it difficult to cope with the multi-component complex systems commonly existing in practical applications. In recent years, researchers have successfully constructed optical fiber sensors for the simultaneous detection of multiple parameters by designing novel fiber structures such as multi-channel optical fibers [28], microstructured optical fibers [29], and optical fiber arrays [30], developing strategies such as multi-site detection and segmented detection [31,32], and adopting advanced signal multiplexing technologies such as wavelength-division multiplexing [33], time-division multiplexing [34], and space-division multiplexing [35]. A variety of optical fiber multi-analyte biochemical sensors have been developed [36,37], which can finally realize the simultaneous detection of multiple biochemical substances.

Among the diverse application fields, liquid biopsy is particularly highlighted as a high-value scenario, where the multiplexed detection of tumor biomarkers has gained growing momentum and exemplifies the translational potential of this technology. Recent comprehensive assessments of photonic sensors for liquid biopsy have emphasized that no single photonic platform is universally optimal. Each technology offers distinct strengths depending on sensitivity, detection limit, and system integration requirements. In this broader landscape, optical-fiber-based sensors offer complementary advantages in miniaturization, remote operation, and multiplexing capability, making them competitive candidates for both liquid biopsy and other translational applications.

**Figure 1:**
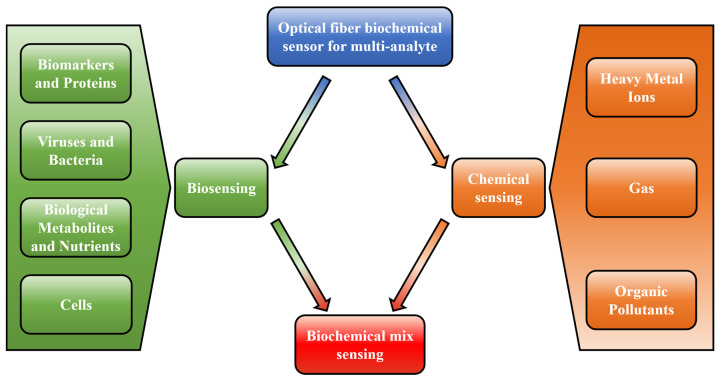
Schematic roadmap.

This review provides a systematic overview of recent progress in optical fiber multi-analyte biochemical sensors for the simultaneous detection of various targets. These sensors are defined herein as detection systems where the optical fiber serves as the primary sensing element, responsible for both light guidance and signal transduction through mechanisms such as surface plasmon resonance, fluorescence, interference, grating coupling, or evanescent wave absorption. Their implementations range from simple chemically functionalized fiber probes and refractive index transducers to more integrated lab-on-fiber or lab-in-fiber platforms that incorporate microfluidics or on-fiber signal processing. Although these implementations differ in technological maturity and integration level, from basic sensing probes to fully self-contained analytical instruments, they all share the optical fiber as the common enabling component and operate under analogous optical transduction principles, which justifies the collective use of the conventional term “optical fiber sensor” throughout this review. The review classifies and discusses these sensors in detail according to the types of detection targets, focusing on working principles, sensitive materials, structural designs, detection methods, and key performance indicators. Figure 1 presents a schematic roadmap of the review. The simultaneous detection capability of optical fiber sensors is naturally suited to the demands of liquid biopsy, where multiple low-abundance biomarkers must be profiled from limited sample volumes. The term “multi-analyte” requires clarification at the outset. In the fiber-optic sensing literature, “multi-analyte”, “multi-parameter”, “multi-channel”, and “multi-component” are frequently conflated but are not synonymous. The present review uses “multi-analyte” for multiple biochemical targets, while “multi-parameter” and “multi-component” denote mixed physical and mixed chemical quantities, respectively. Because many of the systems discussed measure several parameters at once, and the line between biochemical and physicochemical detection is not always clear, a broad scope is adopted: any fiber-based system that detects at least two analytes or parameters relevant to biochemical analysis is included, irrespective of the underlying mechanism. This inclusive logic brings gas analyzers and photonic crystal fiber designs into the same framework as affinity-based sensors. What remains constant is the optical fiber as the core element, not the recognition chemistry. Finally, a comprehensive evaluation of the overall development status and an outlook on future research directions are provided, so as to offer systematic and valuable references for researchers in related fields.

This review covers fiber-based multi-analyte detection studies from approximately 2000 to the present. The literature selected reflects the studies we consider most representative of each analyte category, with emphasis on works that demonstrate clear multiplexing capability and provide sufficient performance data for comparison. Previous reviews in the field have largely been organized around specific sensing mechanisms, such as SPR, fiber gratings, or interferometry, or have focused on particular application domains, including food safety monitoring, biomedical diagnostics, or environmental sensing. While these contributions are valuable, a comprehensive overview that spans all analyte types and examines optical fiber multi-analyte sensors from a unified perspective has been lacking. In particular, no existing review has systematically compared how different sensor architectures and signal multiplexing strategies perform across such a wide range of targets, from biomolecules and pathogens to gases, ions, and cells. The present review aims to fill this gap by providing a systematic classification based on analyte type, while also incorporating mechanistic observations to reveal the design logic behind the reported systems.

Beyond analyte-based classification, the underlying technology of optical fiber multi-analyte sensing can be examined from three interrelated dimensions: the optical transduction mechanism, the structural design of the fiber probe, and the signal multiplexing scheme. Commonly employed transduction mechanisms include surface plasmon resonance, fluorescence, interferometry, grating coupling, Raman scattering, and photoacoustic effects. Probe architectures range from single-channel fibers with site-specific modification to multi-core fibers, fiber arrays, hollow-core fibers and microstructured fibers. Signal multiplexing is typically realized through wavelength-division, time-division, or space-division multiplexing, as well as spectral encoding. These dimensions are not mutually exclusive, and most practical systems combine multiple strategies. Rather than structuring this review around these mechanisms, organization by analyte type is adopted to emphasize application relevance, with mechanism-level observations incorporated to reveal the design logic behind the reported systems.

A final clarification. The literature covered in this review includes both experimental studies and numerical simulations. Two distinct levels of value can be identified in these two categories of work. Experimental work that has been validated in buffer, serum, water, or other relevant matrices is discussed as demonstrated sensing performance. Numerical simulations are included for a different reason: they explore design possibilities and provide theoretical sensitivity limits that may inform future experimental work.

## 2. Optical Fiber Multi-Analyte Biochemical Sensors for Biosensing

### 2.1. Detection of Multiple Biomarkers and Proteins

As the most common detection targets in medical diagnosis, biomarkers and proteins can effectively show human health status [38]. In most cases, the analysis of multiple such substances is required to achieve more accurate diagnosis. There have been many reports on optical fiber biochemical sensors for detecting a single biomarker or protein [39,40,41,42], and they are gradually developing towards multi-analyte detection. At present, optical fiber multi-analyte biochemical sensors capable of recognizing multiple biomarkers and proteins simultaneously can realize detection by measuring optical intensity, fluorescence signal, and phase. The sensor performances are listed in Table 1.

**Figure 2:**
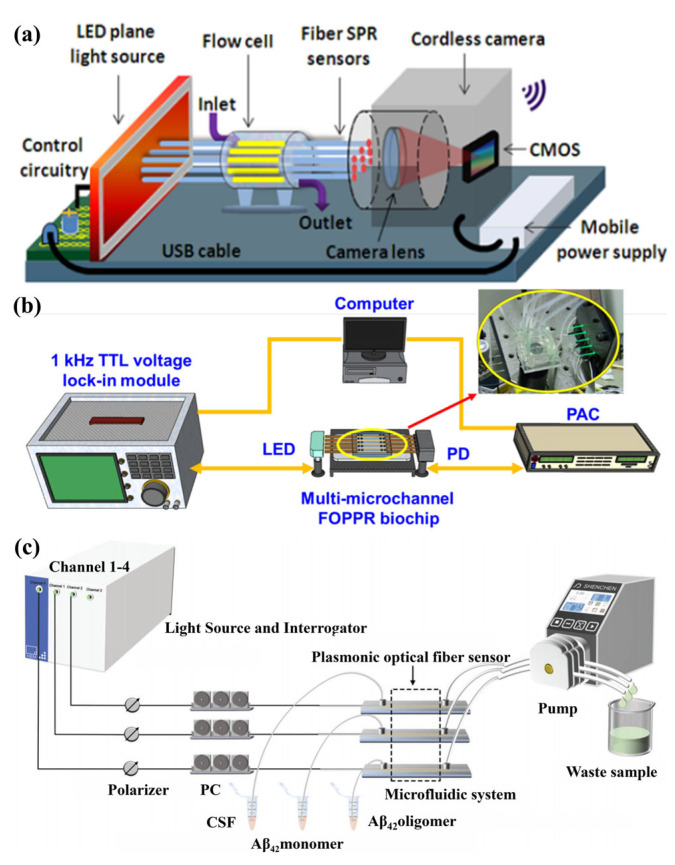
(**a**) Compact multi-channel optical fiber surface plasmon resonance (SPR) biosensor [43]; (**b**) novel multi-microchannel biochip FOPPR sensor [44]; (**c**) TFBG-SPR optical fiber biosensor [46].

The binding of biomarkers and proteins to corresponding sensitive substances on optical fiber multi-analyte biochemical sensors affects the transmission light intensity, reflection light intensity, resonance light intensity, and interference light intensity. The detection of these light intensities enables the simultaneous detection of multiple biomarkers and proteins. In 2015, Liu et al. developed a compact multi-channel optical fiber surface plasmon resonance (SPR) biosensor based on fiber surface coating (Figure 2a) [43], which can be used for real-time, label-free, and quantitative detection of multi-analytes. The real-time detection of immunoglobulin G and concanavalin A was realized by measuring the SPR transmission light intensity. In 2020, Chiang et al. developed a novel multi-microchannel biochip fiber-optic particle plasmon resonance (FOPPR) sensor integrating a photoelectric module (Figure 2b) [44], a phase-locked module, and an integrated platform, which can simultaneously detect streptavidin and anti-dinitrophenyl antibody through the transmission light intensity change generated by SPR. In 2021, Xu et al. developed an all-fiber Fresnel reflection-based microfluidic optical fiber biosensor [45]. After functionalizing specific antibodies at the end of a multimode fiber, the rapid real-time detection of anti-SARS-CoV-2 IgM/IgG was realized by measuring the reflection light intensity. In 2024, Zu et al. developed a tilted fiber Bragg grating (TFBG)-SPR optical fiber biosensor (Figure 2c) [46]. A sensing probe was prepared by functionalizing antibodies on the fiber sensing region. Combined with a microfluidic chip, the ultrasensitive detection and discrimination of Alzheimer’s disease biomarkers Aβ42 monomer/oligomer were achieved by measuring the resonance peak intensity. In 2025, Zhakypbekova et al. developed a semi-distributed interferometer optical fiber biosensor combining enhanced backscattering fiber and fiber Bragg grating [47]. Based on silanization and surface antibody functionalization, the sensing probe realized the simultaneous detection of oral cancer biomarkers interleukin-6 (IL-6) and interleukin-8 (IL-8) by measuring interference intensity.

Despite the common intensity-based readout, these configurations differ in how the signal is generated and isolated from the background. SPR and FOPPR measure transmission loss and are straightforward to implement but are susceptible to bulk refractive index fluctuations. TFBG-SPR reads a resonance dip, offering better background rejection. Fresnel reflection is optically the simplest but relies critically on end-face quality. The interferometric approach distributes sensing sites along the fiber at the cost of more complex interrogation.

**Figure 3:**
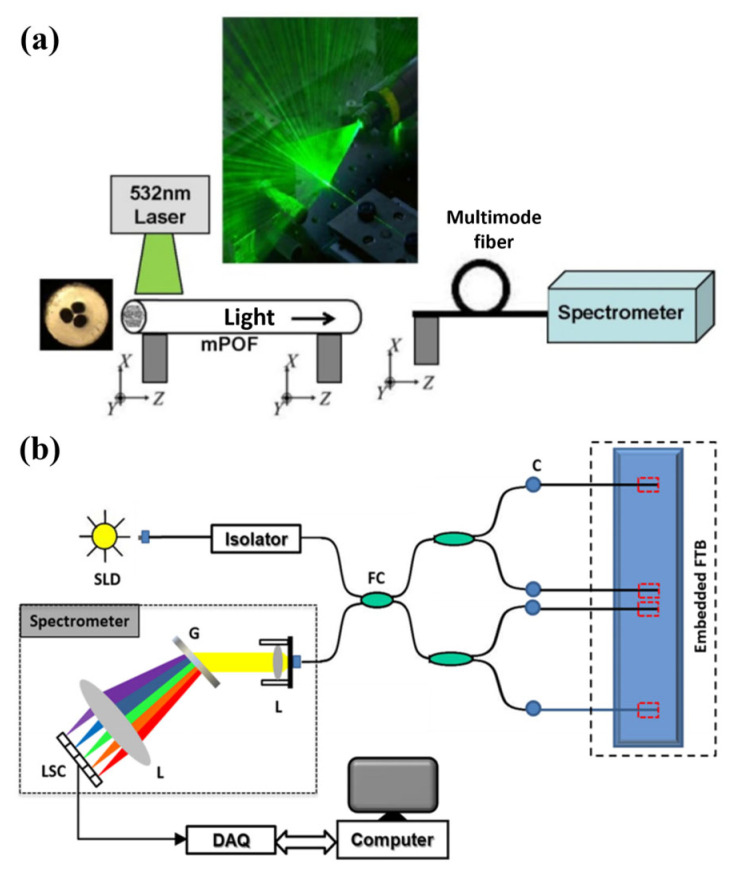
(**a**) Multi-analyte selective optical fiber biosensor fabricated in series within a single microstructured polymer optical fiber [50]; (**b**) distributed optical fiber tip biosensor [52].

Fluorescence and chemiluminescence can specifically respond to different biomarkers or proteins, and the simultaneous detection of multiple biomarkers or proteins can be realized by measuring the change in fluorescence signals. In 2000, Anderson et al. developed a portable and automated optical fiber biosensor (RAPTOR) based on four polymer fiber probes [48]. It could simultaneously detect up to four targets through adsorbing sensitive substances and fluorescence immunoassay, realizing the rapid screening of four biological warfare agents including bacillus globigii, ricin, francisella tularensis, and staphylococcal enterotoxin B by measuring fluorescence intensity. In 2006, Tang et al. developed a tapered optical fiber biosensing probe with specific antibodies immobilized on the surface through an avidin–biotin bridge, forming a fluorescence-mediated optical fiber multi-analyte immunosensing system for the rapid diagnosis and prognosis of cardiovascular diseases (CVDs) [49]. By measuring fluorescence intensity, two major categories of biomarkers, including anticoagulants (protein C, protein S, antithrombin III, plasminogen) and cardiac markers (B-type natriuretic peptide, cardiac troponin I, myoglobin, C-reactive protein), could be quantified simultaneously. In 2013, Emiliyanov et al. demonstrated a multi-analyte selective optical fiber biosensor fabricated in series within a single microstructured polymer optical fiber (mPOF) (Figure 3a) [50]. Using the inertness of TOPAS polymer and photochemical linking technology, different biorecognition layers were immobilized in different segments of the fiber, enabling the detection of α-Streptavidin and anti-C-reactive protein through fluorescence signal changes. In 2024, Zhao et al. constructed a novel fork-DNA-Opt sensor on the surface of gold nanoparticle (AuNP)-modified optical fiber [51]. The sensing region was modified with a walker strand and 6-carboxyfluorescein-labeled fork-type track. The simultaneous detection of miRNA-21 and miR-375 was realized by measuring fluorescence and chemiluminescence signals. These fluorescence-based methods show a clear progression in integration density. The RAPTOR system uses separate fiber probes for each target, which is straightforward but limits miniaturization. The tapered fiber and mPOF designs integrate multiple recognition sites on a single probe, reducing the footprint. The DNA nanomachine approach achieves the lowest detection limits but introduces the most complex assay protocol.

In addition to measuring optical intensity and fluorescence signals, the simultaneous detection of multiple biomarkers and proteins can also be realized by measuring phase and resonance wavelength shift. In 2023, Chirvi et al. developed a distributed optical fiber tip biosensor probe based on Fabry–Pérot (FP) cavity functionalization for the simultaneous measurement of label-free biomolecular interactions at multiple positions in a single data stream (Figure 3b) [52]. Using coherent multiplexing technology to detect phase and distinguish data from different channel sensors, the detection of multiple biomarkers and proteins such as anti-IgG, streptavidin, anti-laminin, anti-goat IgG, and anti-rabbit IgG was realized. In 2024, Li et al. developed a multiplexed dual optical microfiber sensor [53]. Two microfibers with similar diameters were cascaded, and their surfaces were modified with gold nanorods (GNRs) and gold nanobipyramids (Au NBPs), respectively, to excite strong localized surface plasmon resonance (LSPR). The detection of prostate-specific antigen and long noncoding RNA prostate cancer antigen 3 was realized by measuring resonance wavelength shift. Both shift from intensity to wavelength or phase readout, offering better stability against source fluctuations. The distributed FP tip uses coherent multiplexing, which is conceptually powerful but demands sophisticated phase interrogation. The dual-microfiber LSPR sensor is simpler to implement but requires two separate functionalization steps.

From a mechanistic standpoint, most of these sensors rely on either intensity-based SPR or fluorescence readout, with wavelength-interrogated configurations offering better resolution for multiplexed detection.

At present, optical fiber multi-analyte biochemical sensors show significant advantages in detection sensitivity, multi-channel detection capability, and structural miniaturization in biomarker and protein detection, and can realize detection through various methods. Especially in the detection of cancer, cardiovascular disease, Alzheimer’s disease, and infectious disease markers, they demonstrate the potential to replace traditional immunoassays. However, for real-time clinical monitoring applications, further verification is required in more biological sample tests, with emphasis on sensor consistency and long-term stability, to ensure repeatable and reliable detection.

### 2.2. Detection of Multiple Viruses and Bacteria

As one of the greatest threats to human health, viruses and bacteria are diverse and widely distributed in the human living environment [54], making them important targets in biochemical detection. Optical biochemical sensors can achieve specific detection for a single virus or bacterium [55,56,57,58]. Based on this, research is being conducted in the direction of detecting multiple viruses or bacteria. In recent years, optical fiber multi-analyte biochemical sensors have been able to realize the simultaneous detection of more than two viruses and bacteria by measuring wavelength shift and fluorescence signal, showing good development potential. The sensor performances are listed in Table 2.

**Figure 4:**
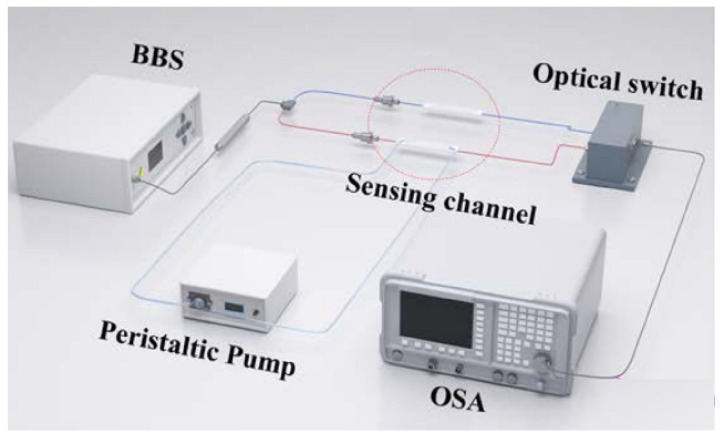
Label-free dual-channel microfiber sensor [59].

Viruses and bacteria can change the effective refractive index of the optical fiber cavity through adsorption and specific binding, thereby affecting the resonance wavelength and the wavelength of the confinement loss peak. The simultaneous detection of multiple viruses and bacteria can be realized by measuring the shift in these two wavelengths. In 2025, Tong et al. proposed a label-free dual-channel microfiber sensor based on silanization covalent bonding (Figure 4) [59]. Using the strong evanescent field of microfiber and the principle of specific immune recognition, the simultaneous detection of influenza A antigen and SARS-CoV-2 antigen was realized by measuring the wavelength shift in interference spectra. Zulkeflee et al. proposed a cascaded single-mode tapered fiber (cascaded SMTF) optical fiber biosensor [60]. Antibody modification was completed through silanization and functionalization respectively, and the simultaneous, label-free, and highly sensitive detection of dengue virus type II E protein and SARS-CoV-2 S protein was realized by measuring resonance wavelength shift. Deepti et al. developed a photonic crystal fiber (PCF)-based multi-analyte optical fiber biosensor [61]. The three-core structure with multiple analyte flow channels facilitates the mode coupling between the liquid to be tested and silica. The detection of four pathogens in water (*Vibrio cholerae*, *Bacillus anthracis*, *Escherichia coli*, *Enterococcus faecalis*) was realized by measuring the wavelength shift in confinement loss peaks. In 2026, Yan et al. cascaded two tapered fibers with different diameters, and fixed specific primers for hepatitis B virus and hepatitis C virus on their surfaces to construct an in-line cascaded tapered microfiber sensor [62]. The label-free simultaneous detection of hepatitis B virus and hepatitis C virus DNA was realized by measuring the wavelength shift in interference spectra. All four detect pathogens through refractive index changes, but with different multiplexing configurations. The cascaded tapered fibers produce distinct interference patterns from different diameters, the PCF design uses separate cores as independent channels, and the in-line tapered microfiber adds enzymatic amplification for improved sensitivity, at the cost of stricter temperature control.

In addition to wavelength modulation, the change in fluorescence signal can also realize the simultaneous detection of multiple viruses or bacteria. In 2013, Ohk et al. developed a multiplex optical fiber immunosensor based on evanescent wave fluorescence [63]. A sensing probe was prepared by modifying biotinylated polyclonal antibodies on the fiber surface, enabling the simultaneous detection of *Listeria monocytogenes*, *Escherichia coli* O157:H7, and *Salmonella enterica* in ready-to-eat meat samples by measuring fluorescence signals. In 2025, Guo et al. developed a portable and intelligent optical fiber fluorescence detection system combining a CRISPR/Cas12a system [64], a single-stranded DNA fluorescent probe (ssDNA-FQ) with fluorescence quencher, and an RBF neural network (RBFNN). The real-time high-throughput detection of African swine fever virus (ASFV) and *Salmonella* was realized by measuring fluorescence signals. Both use fluorescence readout, but the CRISPR/Cas12a-based system achieves significantly lower detection limits by combining specific recognition with neural-network-assisted data processing, albeit at the cost of more complex reagent handling compared with the conventional immunoassay format.

The reported sensors predominantly employ wavelength shift detection through tapered fiber interferometers or photonic crystal fiber designs, with fluorescence-based methods used mainly in immunoassay formats.

Existing optical fiber multi-analyte biochemical sensors can simultaneously detect a variety of pathogenic microorganisms, with the advantages of high sensitivity, multi-functional integration, and intelligence, enabling rapid detection of pathogenic microorganisms, respiratory viruses, and bacteria in water. However, relevant research is still limited, and more practical and complex sample tests are needed to verify the accuracy and reliability of detection. The repeatable fabrication capability and consistency of sensors also need supplementary verification.

### 2.3. Detection of Multiple Biological Metabolites and Nutrients

Biological metabolites and nutrients are important parameters for evaluating biological status and can effectively reflect the health of organisms [65]. Optical fiber sensing technology has developed in detecting a single biological metabolite or nutrient [66,67,68,69], and optical fiber biochemical sensors for the detection of multiple biological metabolites or nutrients are also being developed. Existing optical fiber multi-analyte biochemical sensors mainly realize the simultaneous detection of multiple biological metabolites and nutrients by measuring wavelength shift, improving detection efficiency. The sensor performances are listed in Table 3.

The adsorption and specific binding of biological metabolites and nutrients can change the output spectrum of optical fiber multi-analyte biochemical sensors. The simultaneous detection of multiple biological metabolites and nutrients can be realized by measuring resonance wavelength shift and interference wavelength shift. In 2016, Tabassum et al. reported a multi-analyte sensing probe based on cascaded channel fiber SPR [70]. It was prepared by modifying multi-walled carbon nanotube (MWCNT)–chitosan nanohybrids and Polybrene@ZnO core–shell nanostructures on two different fibers, and could be used for simultaneous detection of vitamin K_1_ and heparin by measuring resonance wavelength shift. In 2022, Zheng et al. reported a plug-and-play reflective optical fiber SPR dual-parameter biosensor [71]. After gold coating and AuNP modification at different positions of the fiber, PMBA and β-cyclodextrin (β-CD) were modified to excite two SPR dips, enabling simultaneous measurement of glucose and cholesterol concentrations by measuring resonance wavelength shift. In 2025, Cheng et al. proposed a dual-channel SPR-based optical fiber biosensor [72]. Using ZnO nanoparticles as a matrix, urease and uricase were immobilized simultaneously, and a Nafion ion-exchange membrane was used to isolate the urea-sensing region to avoid crosstalk. The simultaneous detection of urea and uric acid concentrations was realized by measuring resonance wavelength shift. In 2026, Gong et al. proposed a three-mode optical fiber biosensor based on femtosecond laser micromachining [73]. By integrating MZI, SPR, and fiber Bragg grating (FBG) sensing modes in a microstructured optical fiber (MOF) and modifying ZIF-8/urease and polyacrylic acid/creatininase composite films, the simultaneous monitoring of creatinine, urea, and temperature was realized by measuring resonance wavelength and interference wavelength shift. These designs show an increasing trend in integration: from separate fibers for each analyte, to spatially separated regions on a single fiber, to multiple sensing modalities (MZI, SPR, FBG) on the same fiber. Each step increases integration density but also introduces more challenges in signal separation and crosstalk management.

In addition, fluorescence signal measurement can also be used for the detection of biological metabolites and nutrients. In 2018, Song et al. proposed a FRET-based dual-color evanescent wave all-fiber aptasensor [74]. Using a dual-wavelength laser and optical switch to achieve simultaneous excitation and collection of dual-color fluorescence (Cy5.5 and Alexa 405), the detection of aflatoxin M1 and ochratoxin A was realized by measuring fluorescence signals. In 2026, Yi et al. constructed a nanofiber probe (NFP)-mediated optofluidic dual-laser biosensor (NFP-ODLB) [75]. Complementary DNA (cDNA) of ricin and abrin aptamers was covalently immobilized on NFP to form cDNA-functionalized NFP, enabling the detection of ricin and abrin by measuring fluorescence signals. Both use aptamers as recognition elements, offering advantages over antibodies in thermal stability and small-molecule discrimination. The evanescent wave sensor is simpler in operation, while the nanofiber optofluidic system achieves lower detection limits through more sophisticated fluidic handling.

Current optical fiber multi-analyte biochemical sensors have demonstrated the ability to detect multiple physiologically relevant biomarkers and nutrients simultaneously with high sensitivity and low detection limits, which makes them attractive for multi-index diagnostic assays and dynamic health monitoring. Among the various transduction mechanisms, SPR-based wavelength interrogation remains the dominant approach, whereas fluorescence-based aptasensors offer a promising alternative for small-molecule detection. Despite these advantages, existing studies still face several limitations: the number of real-sample validations is often small, the range of detectable analytes remains narrow, and the level of device integration is relatively low. In addition, sensor-to-sensor consistency and fabrication reproducibility have not been thoroughly validated. These issues warrant further investigation before such sensors can be reliably deployed in practical settings.

### 2.4. Detection of Multiple Cells

In addition to the detection of various biochemical substances, viruses, and bacteria, the detection of human tissue cells, especially cancer cells from different human tissues, has attracted much attention and important medical value [76]. Optical fiber biochemical sensors have made great progress in the field of cell detection [77,78,79,80], and the detection of multiple cells has also been regarded as an important development direction. At present, optical fiber multi-analyte biochemical sensing can realize the simultaneous detection of multiple cancer cells by measuring absorption spectrum, wavelength shift, and confinement loss. The sensor performances are listed in Table 4.

The change in absorbance in an absorption spectrum can effectively reflect the number of cancer cells to achieve detection. In 2020, Singh et al. proposed an LSPR biosensor based on etched multi-core fiber [81]. The sensing performance was enhanced by modifying copper oxide nanofibers (CuO-NFs), graphene oxide, and AuNPs, enabling detection of various cancer cells such as human liver cancer cells HepG2, mouse liver cancer cells Hepa 1-6, human breast cancer cells A549, human lung cancer cells MCF-7, human normal liver cells LO2, and normal canine fibroblasts NCF by measuring absorption spectra.

The detection of cancer cell concentration or quantity can be realized by measuring resonance wavelength shift and confinement loss change. In 2025, Debbarma et al. proposed a novel dual-channel plasmonic biosensor based on a PCF and Ta_2_O_5_/Au double-layer film structure [82], enabling simultaneous detection of adrenal gland cancer cells PC12 and cervical cancer cells HeLa by measuring SPR wavelength shift and confinement loss. Dashtmian et al. designed a D-shaped PCF-based optical fiber SPR biosensor with a gold layer and TiO_2_ layer SPR structure [83], enabling accurate detection of various cancer cells such as skin cancer cells (Basal), cervical cancer cells HeLa, blood cancer cells Jurkat, adrenal gland cancer cells PC-12, and breast cancer cells MDA-MB-231 by measuring resonance wavelength shift and confinement loss. These studies differ fundamentally in their nature. Singh’s etched fiber sensor is experimentally validated, achieving single-digit cells/mL detection limits through absorption measurement. The two PCF-SPR studies are numerical simulations that predict sensitivity based on refractive index changes, awaiting experimental confirmation. The contrast highlights a gap between simulation-based design and experimental validation in cell detection.

Current cell detection relies almost exclusively on refractive index discrimination through SPR or photonic crystal fiber configurations, with absorbance-based readout as a secondary option.

Optical fiber multi-analyte biochemical sensors exhibit high sensitivity and specificity in the detection of multiple cancer cells, enabling simultaneous discrimination and quantification of more than two cancer cells, showing the potential for high-throughput cancer cell detection. However, verification of response time and repeatability is lacking, and differences in detection indicators caused by cell activity still need supplementary verification to ensure detection accuracy.

## 3. Optical Fiber Multi-Analyte Biochemical Sensors for Chemical Sensing

### 3.1. Detection of Multiple Heavy Metal Ions

Heavy metal ions are important pollutants in environmental pollution [84], with diverse types and wide distributions. Excessive heavy metal ions will cause serious ecological damage and water pollution, especially when multiple heavy metal ions coexist. Biochemical sensors developed based on optical fibers for detecting heavy metal ions have been widely reported [85,86,87,88], and optical fiber biochemical sensors capable of detecting multiple heavy metal ions have been continuously emerging in recent years. Existing optical fiber multi-analyte biochemical sensors can realize the detection of multiple heavy metal ions by measuring wavelength shift, electrochemical signal, and amplitude. The sensor performances are listed in Table 5.

**Figure 5:**
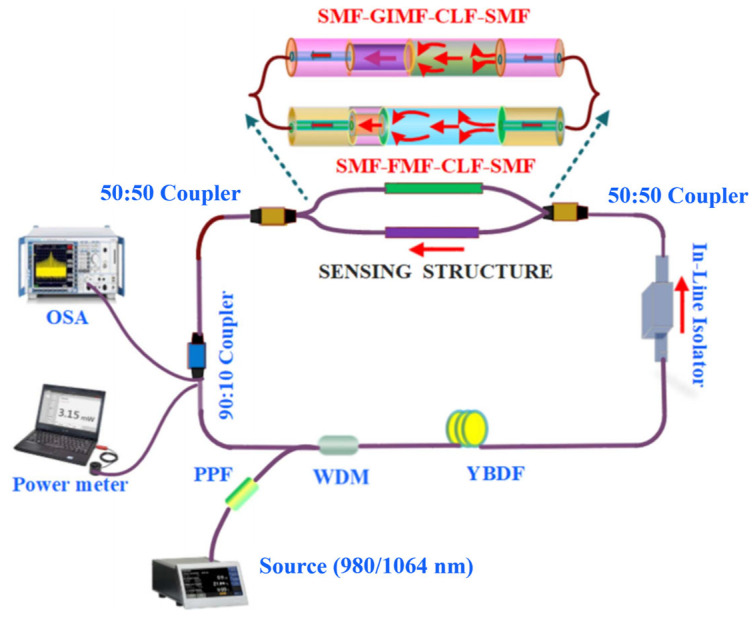
Multi-channel optical fiber transmission sensor based on the MMI effect [90].

The specific adsorption and reaction of heavy metal ions can affect the resonance wavelength of optical fiber grating, fiber laser, and optical fiber surface plasmon sensors. The simultaneous detection of multiple heavy metal ions can be realized by measuring resonance wavelength shift. In 2024, Liu et al. proposed an optical fiber grating biochemical sensor based on phase-shifted long-period fiber grating (PS-LPFG) functionalized with polyacrylic acid and chitosan-sensitive films [89], enabling specific detection of Ni^2+^ and Zn^2+^ in water by measuring resonance wavelength shift. Anuradha et al. proposed a multi-channel optical fiber transmission sensor based on the multimode interference (MMI) effect (Figure 5) [90], integrated into a fiber ring laser (FRL). The multi-channel configuration includes SMF-CLF-GIMF-SMF (SCGS) and SMF-CLF-FMF-SMF (SCFS) structures for heavy metal ion detection, enabling simultaneous detection of Cu^2+^ and Zn^2+^ by measuring laser wavelength shift. In 2026, Yin et al. designed a multiplexed plasmonic sensing platform based on MOF [91]. A sensing probe was prepared by precisely depositing a MoS_2_ interlayer on a gold film and combining it with a specific ion-imprinted polymer (IIP) for functionalization, enabling parallel detection of Cu^2+^ and Ni^2+^ with temperature compensation by measuring resonance wavelength shift. These three sensors achieve ion selectivity through different strategies. PS-LPFG with functionalized polymers relies on chelation. The fiber ring laser enhances sensitivity through sharp laser linewidth. MoS_2_ with ion-imprinted polymers provides the highest specificity through recognition sites created during polymerization, but at the cost of more complex preparation.

In addition to pure resonance wavelength shift measurement, electrochemical current signals can also be used for parallel detection. In 2024, Peng et al. proposed an SPR–electrochemical dual-mode optical fiber sensing probe based on a multimode fiber–single mode fiber (MMF–SMF) reflection probe [92], which can excite SPR for optical sensing and act as an electrochemical working electrode. The simultaneous and real-time trace detection of Pb^2+^ and Cu^2+^ in mixed solution was realized by measuring resonance wavelength shift and stripping current. The dual-mode design combines optical SPR with electrochemical stripping voltammetry, achieving femtomolar-level detection limits. This is a departure from purely optical methods, offering cross-validation between two independent signals and the ability to detect trace metals through pre-concentration, but requires careful electrode design and signal processing.

In recent years, methods for the simultaneous detection of multiple heavy metal ions by measuring resonance peak amplitude have also emerged. In 2025, Yan et al. developed an optical fiber sensing platform for heavy metal ions based on the AgNPs@Cu-TCPP(Pt) composite plasmonic structure [93]. A highly sensitive sensing platform with composite plasmonic structure was constructed by successively modifying gold film, 2D MOF material Cu-TCPP(Pt), and AgNPs on the surface of TFBG, enabling real-time, multi-component detection of Pb^2+^, Cd^2+^, and Hg^2+^ by measuring resonance peak amplitude. Unlike the wavelength-interrogated systems above, this sensor uses amplitude as the readout, which allows faster response as it does not require spectral scanning. The composite plasmonic structure with AgNPs and 2D MOF enhances sensitivity, but the fabrication complexity may limit reproducibility.

The integration of SPR with electrochemical readout or functionalized optical fiber grating structures represents one of the most distinctive trends in this category, with fiber interferometers offering an alternative design route for such sensors.

Optical fiber multi-analyte biochemical sensors exhibit high sensitivity, a low detection limit, and high selectivity in the parallel detection of multiple heavy metal ions, overcoming the bottleneck of single-ion detection. However, there are still problems to be optimized, such as limited types of detected ions, treatment of environmental interference, detection stability, and reusability. The treatment of environmental interference needs to be improved, and the number of detectable ion species under anti-interference conditions is still limited.

### 3.2. Detection of Multiple Gases

The gas sensing section covers a wide range of detection principles, many of which are spectroscopic. The reason for including them is twofold. The gases discussed here are not physical targets but biochemically and environmentally relevant species routinely measured in breath analysis, food quality assessment, and environmental monitoring. In addition, the multiplexing techniques developed in gas sensing have influenced liquid-phase biochemical sensing more broadly. This section is therefore included not only to cover an application area but also to document technological strategies that are relevant across different analyte types.

As important detection parameters in industrial production, food manufacturing, and other fields [94], gases require strict detection and control, mostly for component detection of mixed gases. Many studies have been conducted on optical fiber multi-analyte biochemical sensors in the field of gas detection. Optical fiber gas sensors have been popularized and commercialized [95,96,97,98], and optical fiber biochemical sensors for the detection of multiple gases are gradually becoming the mainstream research direction. The simultaneous detection of multiple gases can be realized by measuring optical intensity, wavelength shift, amplitude, fluorescence signal, absorbance, and other methods. The sensor performances are listed in Table 6.

**Figure 6:**
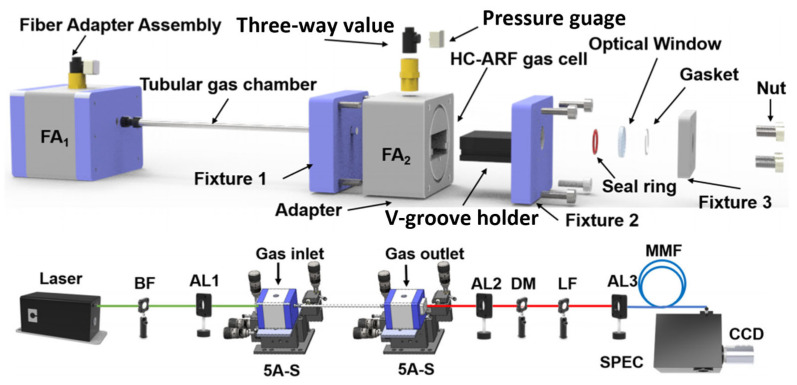
Gas detection system based on HC-ARF enhanced Raman spectroscopy [103].

The simultaneous detection of multiple gases by optical intensity is mainly realized by measuring output light intensity, transmission light intensity, and Raman scattering intensity. In 2014, Shobin et al. coated MWCNT functionalized with isobutyl methyl ketone (IBMK) on cladding-removed polymethyl methacrylate (PMMA) fiber by dip-coating to form an intensity-modulated optical fiber gas sensor [99], enabling detection of methanol and ethanol vapor by measuring output light intensity. In 2017, Gui et al. proposed an optical multi-component gas optical fiber sensor based on a conjugated interferometer (CI) [100], which can convert the absorption spectrum of the target gas into a conjugated emission spectrum. Combined with a broadband light source, the output light spectrum is precisely matched with the absorption spectrum of the target gas, enabling mixed concentration detection of C_2_H_2_ and NH_3_ by measuring output light intensity. In 2018, Yan et al. prepared an optical fiber sensor for high-temperature multi-component gas sensing using standard silica optical fiber coated with a single functional oxide La_0.3_Sr_0.7_TiO_3_ (LSTO) [101]. Combining two near-infrared optical mechanisms (broadband absorption and hydroxyl thermal emission) and principal component analysis (PCA), the selective identification and concentration monitoring of H_2_, CO_2_, CH_4_, and CO at 800 °C were realized by measuring near-infrared transmission light intensity. In 2025, Wan et al. developed a Raman spectroscopy gas detection system based on hollow-core anti-resonant fiber (HC-ARF) [102]. A multi-stage spatial filtering method can suppress background fluorescence noise with uneven spatial distribution, enabling highly sensitive detection of ^13^CO_2_ and ^12^CO_2_ in air by measuring Raman scattering intensity. Zhu et al. developed a gas detection system based on HC-ARF enhanced Raman spectroscopy (Figure 6) [103]. By designing a dual-lens signal collection module combined with small-core multimode fiber and a CCD row-selective integration strategy, system background noise was effectively suppressed, enabling detection of ^12^CO_2_, ^13^CO_2_, and various hydrocarbon gases by measuring Raman scattering intensity. These intensity-based methods cover a wide range of physical principles, from simple refractive index changes and conjugated interferometry to broadband absorption with PCA and hollow-core fiber Raman scattering. The common trend is toward using more sophisticated signal processing to extract compositional information from relatively simple optical measurements.

**Figure 7:**
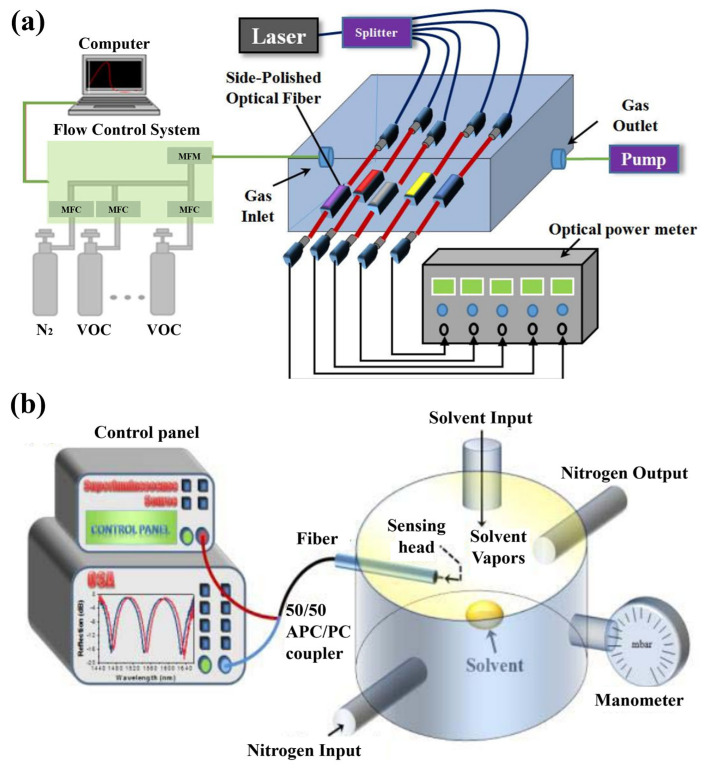
(**a**) Multi-array volatile organic compound (VOC) gas sensor combining side-polished fiber and a dye-coated planar waveguide [104]; (**b**) optical fiber gas sensor formed by two-photon polymerization technology [105].

The simultaneous detection of multiple gases can also be realized by measuring resonance wavelength and interference wavelength shift. In 2014, Lee et al. developed a multi-array volatile organic compound (VOC) gas sensor combining side-polished fiber and a dye-coated planar waveguide (PWG) (Figure 7a) [104]. Different dyes were doped into PVP polymer and coated on five independent side-polished fibers to form sensing films, enabling detection of different VOC gases (ethanol, benzene, dimethylamine, acetic acid, and toluene) by measuring resonance wavelength shift. In 2016, Melissinaki et al. directly laser-wrote a FP micro-resonator on the end face of standard communication fiber using two-photon polymerization technology to form an optical fiber gas sensor (Figure 7b) [105]. Based on physical adsorption and a molecular stacking mechanism, the detection of different chlorinated organic solvent vapors (CH_2_Cl_2_, CHCl_3_, and CCl_4_) was realized by measuring interference spectrum wavelength shift. Both sensors detect VOCs through wavelength shifts, but the selectivity mechanisms differ. Lee’s dye-doped polymers achieve chemical specificity through dye–analyte interactions. Melissinaki’s FP micro-resonator relies on physical adsorption and molecular stacking, which is simpler but offers less discrimination between different chlorinated solvents.

Photoacoustic signal is an important parameter for gas detection, and the simultaneous detection of multiple gases can be realized by measuring the amplitude of photoacoustic signal. In 2016, Mao et al. constructed an all-optical photoacoustic spectrometer (PAS) with a diaphragm-based FP interferometric optical fiber acoustic sensor as the detection unit for multi-gas analysis [106], enabling the simultaneous measurement of five trace gases (H_2_O, CH_4_, C_2_H_2_, CO, and CO_2_) by measuring photoacoustic signal amplitude. In 2023, Zhang et al. designed a fiber ring laser intracavity dual-component gas sensor based on PAS [107]. Using time-division multiplexing (TDM) technology to control C-band and L-band erbium-doped fibers in combination with FBG attached to a piezoelectric transducer (PZT) for wavelength modulation, the time-division detection of C_2_H_2_ and CO_2_ was realized by measuring photoacoustic signal amplitude. In 2024, Zhang et al. designed a compact dual T-type resonant optical fiber photoacoustic sensor including two T-type photoacoustic units and a cantilever-based optical fiber acoustic sensor [108], enabling simultaneous detection of CH_4_ and C_2_H_2_ by measuring photoacoustic signal amplitude. Wu et al. reported a multi-mechanism resonance-enhanced optical fiber photoacoustic sensor combining the resonance enhancement of a T-type resonator and silicon cantilever [109], enabling simultaneous detection of CH_4_ and C_2_H_2_ by measuring photoacoustic signal amplitude. These studies show the evolution of photoacoustic multi-gas detection: from single-gas systems, to time-division multiplexing of two gases, to dual-T-type resonant structures, and finally to multi-mechanism resonance enhancement. Each step improves sensitivity and multiplexing capacity at the cost of increasing system complexity.

**Figure 8:**
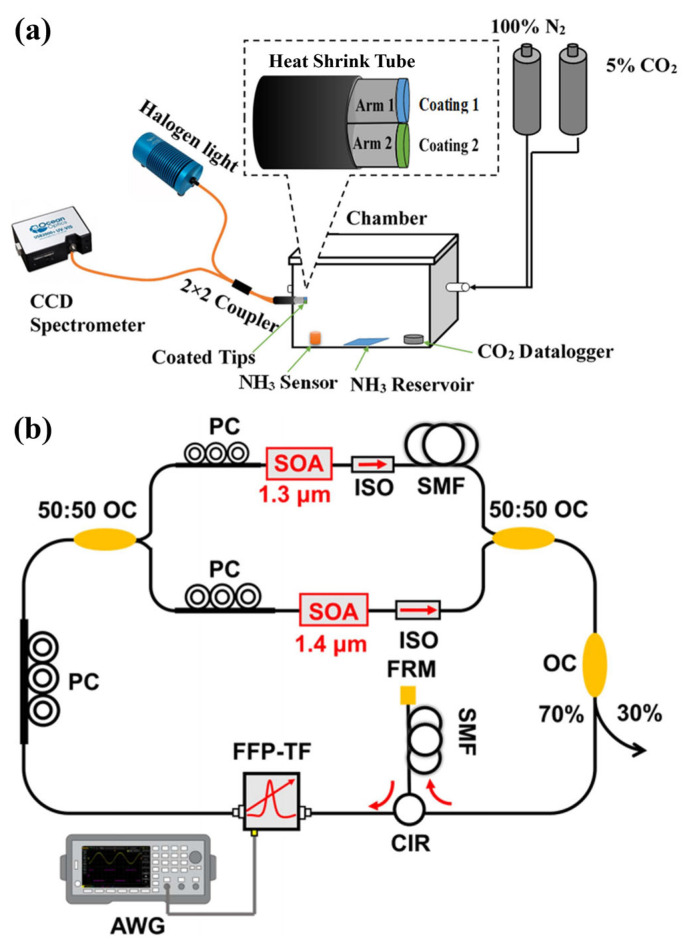
(**a**) Multi-parameter optical fiber gas sensor [110]; (**b**) gas sensor based on broadband Fourier domain mode-locked fiber laser [113].

Different gases have different absorption spectra, and the simultaneous detection of multiple gases can also be realized by measuring absorbance at different bands of absorption spectra. In 2020, Liu et al. prepared a multi-parameter optical fiber gas sensor by coating thymol blue and tetraphenylporphyrin tetrasulfonic acid hydrate on two ends of a 2 × 2 fiber coupler via the sol–gel method, respectively (Figure 8a) [110], enabling simultaneous detection of NH_3_ and CO_2_ by measuring absorption spectra. Xu et al. used a dual-wavelength mode-locked fiber laser as a dual optical comb source combined with a multi-pass absorption unit to form an optical fiber gas sensor [111], enabling simultaneous detection of C_2_H_2_ and NH_3_ by measuring absorption spectra. In 2024, Liu et al. proposed an optical fiber gas sensor based on wavelength scanning spectroscopy using a free-running fiber laser frequency comb and phase-shifted fiber Bragg grating (PS-FBG) [112]. Wavelength scanning and modulation were realized by pasting PZT on PS-FBG, and a Gaussian process regression algorithm was used for spectrum recovery for calibration-free gas sensing, enabling multi-channel detection of multiple gases such as CO_2_ and NH_3_ by measuring absorption spectra. In 2025, Shi et al. developed a gas sensor based on a broadband Fourier domain mode-locked fiber laser (Figure 8b) [113]. Combined with a multi-pass absorption unit, high-speed and real-time measurement of CO_2_, CH_4_, C_2_H_2_, and H_2_O was realized by direct absorption spectrum detection. These absorption-based systems differ in versatility. Chemical indicator methods are simple and compact but limited to gases with suitable indicators. Spectroscopic methods using multi-pass cells offer broader applicability, detecting any gas with distinct absorption features without changing hardware. The frequency-comb-based system achieves the highest sophistication with calibration-free operation.

**Figure 9:**
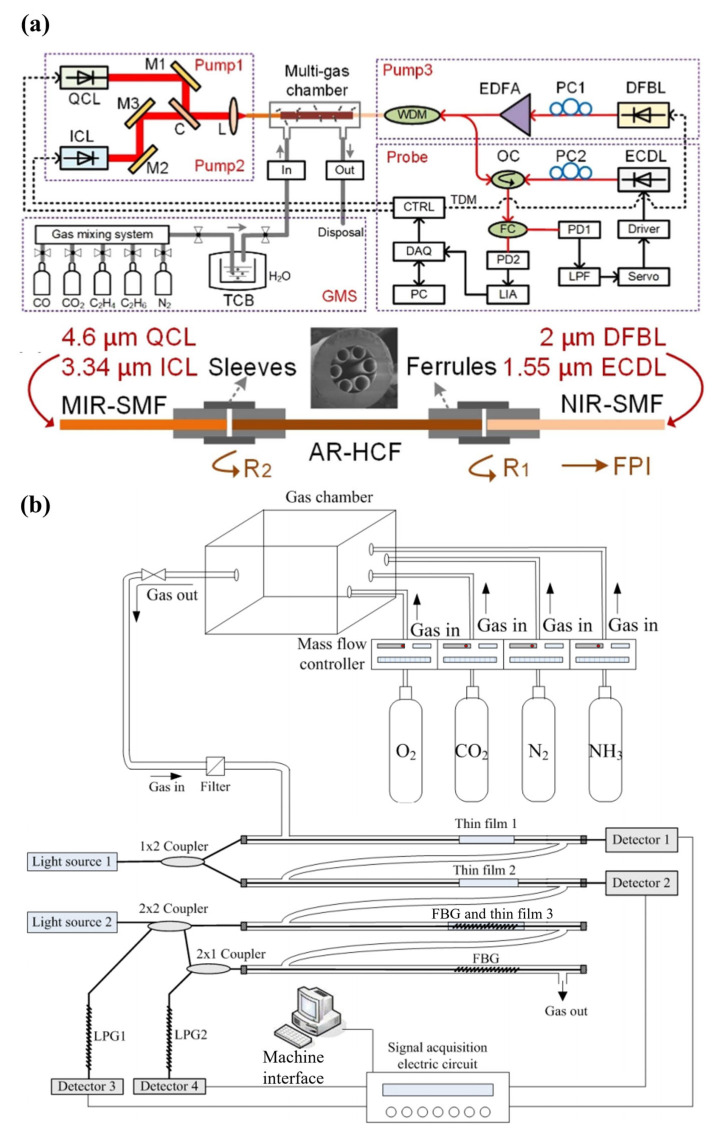
(**a**) Multi-component optical fiber gas sensing system based on mid-infrared PTS [114]; (**b**) novel fiber array gas sensor with temperature compensation [115].

In addition, the real-time detection of multiple gases can also be realized by measuring phase and mixed signals (fluorescence signal and output light intensity). In 2024, Zheng et al. reported a multi-component optical fiber gas sensing system based on mid-infrared photothermal spectroscopy (PTS) (Figure 9a) [114]. Using a HC-ARF as a gas cell, the detection of CO, CO_2_, C_2_H_4_, and C_2_H_6_ was realized by measuring photothermal phase modulation with a FP interferometer composed of a near-infrared detection laser. In 2016, Hung et al. developed a novel fiber array gas sensor with temperature compensation (Figure 9b) [115]. Using materials such as Tris(2,2′-bipyridyl) dichlororuthenium(II) hexahydrate, Tris(bipyridine)ruthenium(II) chloride, and 1-hydroxy-3,6,8-pyrene trisulfonic acid trisodium salt, O_2_ and CO_2_ sensors based on fluorescence quenching and NH_3_ and temperature sensors based on FBG and long-period grating structures were prepared to form a fiber array gas sensor, enabling real-time detection of O_2_, CO_2_, and NH_3_ by measuring fluorescence signal and output light intensity. These two represent different integration philosophies. Zheng‘s photothermal system integrates all functions into a single hollow-core fiber platform, offering high sensitivity and spectral specificity. Hung’s fiber array takes a modular approach, combining different sensor types into an array, which is more flexible and easier to maintain but less elegant. The choice depends on whether performance or robustness is the priority.

Gas sensing is mechanistically the most diverse category, encompassing photoacoustic, Raman, absorption, and interferometric principles, often integrated with hollow-core fibers or fiber lasers for sensitivity enhancement.

Optical fiber multi-analyte biochemical sensors have mature detection technologies for multiple gases, with advantages of high sensitivity, a low detection limit, and wide-range real-time detection. They can monitor multiple gases through the integration of multiple detection principles and various signal processing methods, fully demonstrating the prospects and potential of optical fiber sensors in multi-gas detection. However, in most cases, solutions to cross-sensitivity problems are still limited, and the treatment of environmental interference such as humidity needs to be improved. In addition, most existing studies lack tests on sensor stability and repeatable fabrication, which need supplementary verification to ensure reliability in practical engineering applications.

### 3.3. Detection of Organic Pollutants

Organic pollutants are important factors of environmental pollution and major detection targets in biochemical detection [116]. The detection of organic pollutants has always been the main detection target of optical fiber biochemical sensors [117,118,119,120], with the aim of more and more optical fiber biochemical sensors that can be used for detecting multiple organic pollutants. Existing optical fiber multi-analyte biochemical sensors mainly realize the detection of organic pollutants by measuring optical intensity, fluorescence signal, and absorption spectrum. The sensor performances are listed in Table 7.

**Figure 10:**
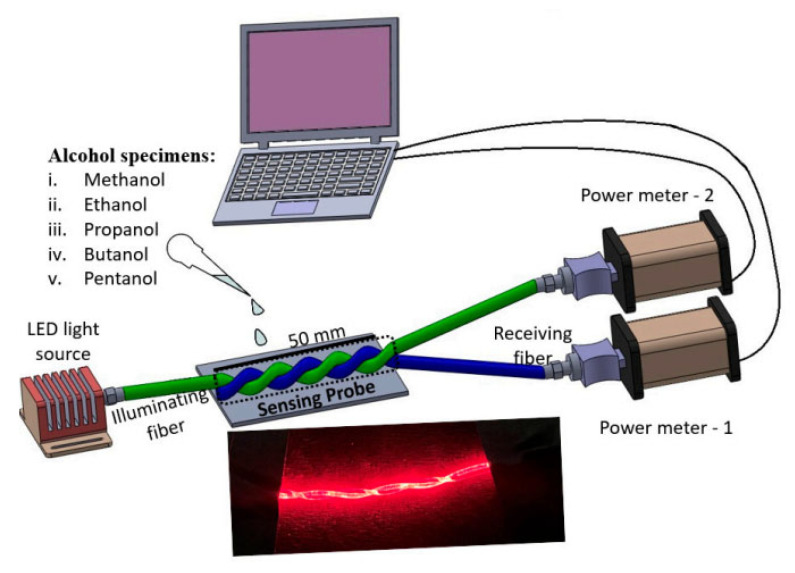
POF alcohol sensor using a simple twisting technique [121].

The simultaneous detection of multiple organic pollutants can be realized by measuring the intensity of coupled optical power. In 2024, Ghaffar et al. prepared a plastic optical fiber (POF) alcohol sensor using a simple twisting technique (Figure 10) [121]. Composed of two twisted POFs for illumination and signal reception respectively, the detection of methanol, ethanol, propanol, butanol, and pentanol was realized based on the change in coupled optical power intensity. In 2025, Li et al. prepared tapered POF by simple heating and stretching, and twisted them together to form a coupled-structure optical fiber alcohol sensor [122]. Based on the principle that coupled optical power changes with external medium refractive index, the detection of ethanol, propanol, butanol, and pentanol was realized by measuring coupled optical power intensity. Both sensors rely on refractive index differences between alcohols to modulate coupling efficiency, without using any selective coating or functionalization. This makes them extremely simple and low-cost, but the lack of chemical selectivity limits them to samples where the analytes are structurally related and their refractive indices are known to differ.

The simultaneous detection of multiple organic pollutants can also be realized by measuring fluorescence signal changes after the combination of different fluorescence-sensitive substances and multiple organic pollutants. In 2019, Song et al. developed a novel dual-color total internal reflection fluorescence biosensor [123]. Two hapten-protein conjugates, BPA-OVA and 2,4-D-OVA, were immobilized simultaneously on a single tapered optical fiber probe, enabling simultaneous quantitative detection of bisphenol A and 2,4-dichlorophenoxyacetic acid by measuring fluorescence signals. In 2020, Long et al. constructed a portable and automated fluorescence microarray biosensing platform [124]. Based on evanescent wave fluorescence immunoassay, optical fiber sensing probes were modified and parallel detection of four channels was realized through time-division multiplexing technology for on-site parallel detection of various environmental pollutants such as microcystin-LR, 2,4-dichlorophenoxyacetic acid, atrazine, and bisphenol A. In 2022, Song et al. developed a dual-fluorescence lab-on-fiber biosensor [125]. Hapten-protein conjugates of two pesticides were co-immobilized on the fiber surface, and acetamiprid antibody and fipronil antibody labeled with Cy5.5 and Alexa fluor 555 were modified on the fiber surface to prepare sensing probes, enabling detection of acetamiprid and fipronil by measuring fluorescence signals. All three use antibody-based specificity with fluorescence readout. The technology has been scaled from two analytes on a single probe to four analytes using microfluidic time-division multiplexing, showing a clear path toward higher throughput while maintaining the selectivity advantage of immunoassays.

In addition, infrared spectrum absorption can also effectively detect multiple organic pollutants. In 2024, Zhao et al. prepared a tapered optical fiber ring sensor by tapering and bending Ge_10_As_30_Se_38_Te_22_ infrared fiber [126]. Based on mid-infrared fiber evanescent wave spectroscopy (MIR-FEWS), the qualitative and quantitative analysis of organic pollutants in water (CH_3_CH_2_OH, C_6_H_5_CH_2_OH, CH_3_CN, and C_6_H_5_CHO) was realized by measuring infrared absorption spectra. Unlike the fluorescence methods above, this approach uses intrinsic mid-infrared absorption as the fingerprint, requiring no functionalization or labeling. The tapered ring geometry enhances evanescent field interaction, but the infrared fiber materials are more expensive and fragile than silica fibers. The key advantage is that any organic pollutant with characteristic absorption can be detected without developing new recognition chemistry.

Among optical fiber sensors for multiple organic pollutant detection, fluorescence intensity and absorption measurements predominate, and evanescent wave excitation is frequently employed at the probe level. Transmission intensity or absorption detection is also available as an auxiliary option.

Existing optical fiber multi-analyte biochemical sensors can simultaneously detect a variety of organic pollutants with high sensitivity, diverse detection types, and good reusability, enabling detection in practical sample environments. However, there is a lack of specific compensation methods for temperature cross-sensitivity and verification of non-specific adsorption during detection. Long-term stability tests also need to be supplemented to ensure long-term storage of sensors.

## 4. Optical Fiber Multi-Analyte Biochemical Sensors for Mixed Biological and Chemical Sensing

The biochemical detection of a single type of target is mostly under ideal conditions. Most practical detection processes need to be carried out under the condition of mixing different types of biochemical substances, so the simultaneous detection of different types of biochemical substances is necessary. Many optical fiber sensors have been able to realize the simultaneous detection of multiple parameters [127,128,129,130], and this design is gradually being used to develop optical fiber biochemical sensors for the mixed detection of biological and chemical substances. Current optical fiber multi-analyte biochemical sensors can realize the simultaneous detection of different types of biochemical substances by measuring wavelength shift and fluorescence signal. The sensor performances are listed in Table 8.

The effective simultaneous detection of different types of biochemical substances can be realized by measuring the shift in multiple resonance wavelengths. In 2024, Gong et al. proposed a single-fiber multi-parameter biochemical sensor based on a triple SPR effect [131]. By modifying pDNA, PAA/CS film, and a PDMS temperature-sensitive polymer with independent SPR signals, the signal crosstalk problem in multi-parameter detection was solved, enabling detection of cDNA, pH, and temperature by measuring resonance wavelength shift. In 2025, Amin et al. proposed a hybrid sensing method based on PCF-SPR. A multi-parameter detection optical fiber biochemical sensor was prepared by coating the external and internal dual-channel structures of asymmetric arc-slot PCF [132], enabling simultaneous detection of SARS-CoV-2 spike RBD glycoprotein and ethyl alcohol concentrations by measuring resonance wavelength shift. In 2026, Yang et al. cascaded and fused a section of hole-assisted triple-core fiber (HATCF) and a section of hole-assisted dual-core fiber (HADCF) to prepare an optical fiber biochemical sensor based on mode resonance coupling [133]. The sensor was prepared by modifying graphene oxide/glucose oxidase and filling polyacrylamide hydrogel, enabling simultaneous detection of glucose concentration, pH, and temperature. These three studies show different ways of integrating biological and physical parameter measurements. The common motivation is that environmental parameters must be monitored alongside biological signals to correct for cross-sensitivity in real applications.

**Figure 11:**
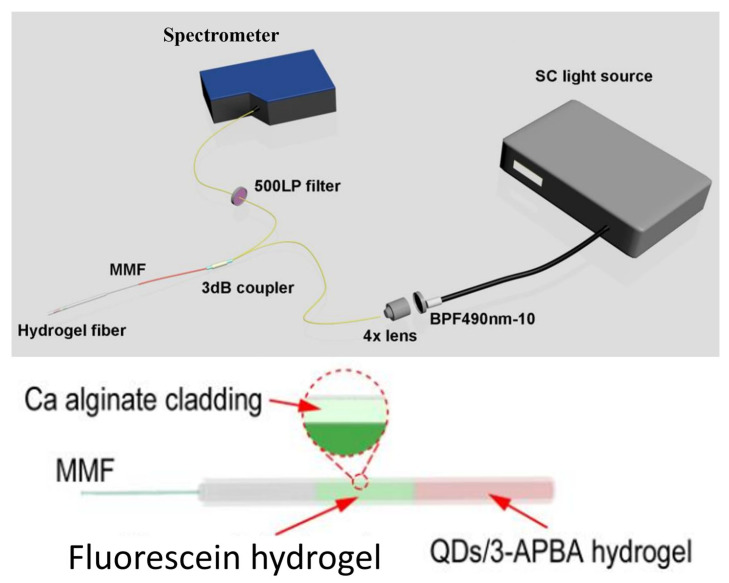
Segmented functionalized hydrogel optical fiber fluorescence sensor [135].

In addition to measuring resonance wavelength shift, multi-type biochemical substance mixed sensing can also be realized by measuring fluorescence signal change. In 1997, Ferguson et al. prepared a multi-analyte imaging optical fiber biochemical sensor by matrix-immobilizing three sensitive substances (acryloylfluorescein, CO_2_-sensitive bicarbonate solution, Ru(Ph_2_phen)_3_^2+^) at the distal end of an optical fiber imaging bundle through photochemical deposition technology [134], enabling simultaneous monitoring of pH, CO_2_, and O_2_ by measuring fluorescence signals, and successfully applied it to the real-time monitoring of the beer fermentation process. In 2023, Li et al. prepared a segmented functionalized hydrogel optical fiber fluorescence sensor (Figure 11) [135]. With p(AM-co-PEGDA) as the core and calcium alginate as cladding, CdTe quantum dots, 3-acrylamidophenylboronic acid (3-APBA), and fluorescein derivatives were doped to prepare sensing probes, enabling simultaneous detection of glucose and pH by measuring fluorescence signals. Both use fluorescent indicators for mixed-parameter sensing but differ in spatial encoding. Ferguson’s imaging fiber bundle allows spatially resolved detection of multiple indicators simultaneously, requiring a camera and image processing. Li’s segmented hydrogel fiber achieves spatial separation along the fiber length through sequential fabrication, offering a simpler optical setup. The imaging approach is more flexible for adding new indicators; the hydrogel approach is more compact.

The mixed-sensing category is characterized by the integration of multiple transduction mechanisms within a single probe, often combining wavelength interrogation with fluorescence or temperature compensation channels.

Optical fiber multi-analyte biochemical sensors can realize the simultaneous detection of multi-type biochemical targets with high sensitivity and rapid response through various detection methods, and are suitable for compensating environmental parameters that cause cross-sensitivity. However, most studies are in the experimental stage, lacking real-sample test verification, long-term stability, and repeatability verification, which cannot guarantee the reliability of sensors in practical applications.

## 5. Discussion

Optical fiber multi-analyte biochemical sensors have attracted considerable attention in recent years for their ability to detect multiple biochemical targets simultaneously. Most of these sensors are realized through either single-site modification of multi-channel fiber probes or multi-site modification of single-channel probes. As reviewed above, they have been successfully applied to a diverse range of analytes, including biomarkers, proteins, viruses, bacteria, metabolites, nutrients, heavy metal ions, gases, organic pollutants, cells, and mixed biochemical substances. Detection is accomplished through various signal transduction methods, such as intensity, fluorescence, phase, wavelength shift, electrochemical current, amplitude, and absorbance measurements. Across the studies surveyed, several trends emerge. SPR and fluorescence-based designs generally yield the lowest detection limits, often reaching the picomolar to femtomolar range for protein and nucleic acid targets. Gas sensing and heavy metal ion detection are the two categories with the most extensive real-sample validation, probably because their measurement matrices are less complex than serum or whole blood. Intensity-based polymer fiber sensors and fiber arrays appear closest to portable deployment, requiring only compact light sources and detectors with minimal supporting optics. A summary of the advantages and disadvantages for optical fiber sensors of each analyte category is provided in Table 9.

A recurring challenge across all categories of optical fiber multi-analyte sensors is crosstalk, which manifests in several distinct forms. Spectral crosstalk, arising from overlapping optical responses such as adjacent SPR dips or fluorescence bands, is managed through orthogonal fluorophores, resonance positioning or spectral unmixing. Spatial-channel crosstalk in multi-core or multi-channel fibers occurs when light couples between adjacent channels, mitigated by physical separation, mode confinement, and channel spacing. Biochemical cross-reactivity, where capture probes bind non-target species, is addressed with high-specificity recognition elements and surface blocking. Mass-transport crosstalk in microfluidic-integrated systems involves analyte diffusion between adjacent zones, controlled by channel geometry, flow rate, or physical barriers. Environmental cross-sensitivity to temperature, pH, ionic strength, or humidity is countered with reference channels, differential measurements, or FBG-based compensation. Not all studies detail these mitigations, and implementation varies with design and application, but recognizing these crosstalk types is essential for evaluating performance and guiding future work.

Beyond crosstalk, broader challenges remain. Real-world matrix effects and environmental interferences such as humidity, temperature fluctuations, and non-specific binding are still inadequately addressed. Validation with clinical or environmental samples is scarce; most studies remain laboratory-based, and reliability for diagnostics, monitoring, or safety testing has yet to be rigorously confirmed. Repeatability and long-term stability are underexplored, with batch consistency and performance under storage or continuous operation needing systematic assessment. The number of simultaneously detectable analytes is limited by available modification sites and channels, and designs often hit spatial, optical, or signal-processing constraints. For cells and mixed biochemical substances, biological variability and system complexity further compromise accuracy, and these effects remain unevaluated. Together, these challenges underscore the gap between laboratory demonstrations and practical applications. Bridging this gap requires not only iterative design improvements but also standardized testing protocols and comprehensive performance criteria.

Microfluidic integration is another important consideration that deserves explicit attention. In many of the reviewed systems, microfluidic chips are used to deliver samples and reagents to the fiber sensing region in a controlled manner in order to isolate different detection channels reduce sample and reagent consumption. The degree of microfluidic integration varies considerably across the studies. Some systems use simple flow cells that direct the sample to the fiber surface, which is straightforward but offers limited control over multiple reagents. Others incorporate more complex channel networks with multiple inlets and outlets, allowing sequential or parallel delivery of different samples and recognition elements to distinct sensing sites. A few designs achieve full integration at the fiber tip, where the fiber itself serves as part of the microfluidic pathway. This variation reflects a broader trend in the field: microfluidics is increasingly viewed not as an add-on but as an enabling component that determines the practical utility of multi-analyte sensors, particularly for applications that require multi-step assays or the handling of limited sample volumes.

Surface functionalization is the molecular basis of specificity in most optical fiber multi-analyte sensors. The strategies reported in the reviewed literature vary widely depending on the target type. Antibodies remain the most widely used recognition elements, particularly for proteins, viruses, and bacteria. For small molecules and metabolites, aptamers offer advantages in thermal stability and ease of synthesis, and they have been used in several fluorescence-based sensors. Enzymes provide a natural route for detecting metabolic substrates such as glucose, urea, and uric acid, with specificity derived from the enzyme–substrate interaction. DNA probes are used for nucleic acid targets, including microRNAs and viral DNA, often combined with amplification schemes for improved sensitivity. For heavy metal ions, ion-imprinted polymers have been employed to create recognition sites through polymerization in the presence of the target ion. Molecularly imprinted polymers serve a similar function for organic pollutants, offering selectivity based on the template molecule. Hydrogels have been used in mixed sensing applications, where they simultaneously immobilize multiple indicators for different parameters. Some studies have also addressed non-specific adsorption through antifouling coatings, though this remains less common. The choice of functionalization strategy determines not only the specificity and sensitivity of the sensor but also its stability, reproducibility, and the complexity of the fabrication process.

Signal processing and data analysis are increasingly important in optical fiber multi-analyte sensing, particularly as the number of simultaneous targets grows. Many of the sensors reviewed here generate complex optical responses, and extracting quantitative multi-analyte information often requires more than simple peak tracking. Spectral deconvolution has been used to separate overlapping resonances or fluorescence bands, allowing individual analytes to be resolved from a composite spectrum. Multivariate regression methods, including partial least squares and principal component regression, have been applied to multi-wavelength or multi-channel data to correlate spectral features with analyte concentrations. Principal component analysis has been used both for dimensionality reduction and for pattern recognition, helping to distinguish responses from different analytes. Neural-network-based approaches have appeared in several recent studies, particularly for intelligent detection and real-time data interpretation. Calibration-free methods, which aim to reduce or eliminate the need for frequent recalibration, have also been explored, often through the use of internal references or self-referencing configurations. These techniques are not uniformly adopted across the reviewed literature, and their implementation varies considerably depending on the sensor type and application. However, the trend is clear: signal processing is moving from an auxiliary step to a core component of multi-analyte sensing, especially as systems become more integrated and field deployable.

A separate set of barriers concerns the translation of these sensors from laboratory demonstration to clinical application. The vast majority of studies reviewed here report performance in buffer solutions, purified protein solutions, or spiked samples. Real clinical matrices, such as serum, plasma, or whole blood, present a much more challenging environment. Biomarkers of early-stage diseases are present at extremely low concentrations, often below the effective detection range of current fiber-based sensors. The high protein content of serum and plasma causes non-specific adsorption and surface fouling, which degrade sensor response over time. Surface functionalization, typically based on antibodies or aptamers, has limited long-term stability and the storage conditions needed to preserve activity are not well standardized. Sample preparation requirements, such as dilution, centrifugation, or extraction, are often mentioned briefly or omitted entirely, but these steps add complexity and limit the practicality of point-of-care deployment. Direct comparison with established clinical methods is difficult: ELISA, PCR, digital PCR, and electrochemical sensors operate on different principles, use different sample types, and report performance in different ways. The same applies to emerging plasmonic chips and bead-based immunoassays. Benchmarking against these standards is essential for clinical acceptance, but few studies have undertaken such comparisons. Regulatory validation, which is required for any clinical diagnostic device, remains a distant goal for most optical fiber multi-analyte sensors. Recognizing these barriers is important not to discourage research but to identify the areas where future work is most needed.

It is also useful to distinguish, in broad terms, which of the reviewed technologies are closer to practical deployment and which remain at an earlier stage. By the criteria of real-sample validation and the availability of prototypes that can operate outside a laboratory setting, intensity-based polymer fiber sensors and fiber arrays are the closest to near-term deployment. They are simple, inexpensive, and already used in some field applications. SPR-based and grating-based fiber sensors have been validated in a range of biological matrices but still rely on laboratory-grade optical interrogation systems, which limits their portability. Hollow-core fiber systems for gas sensing, photoacoustic sensors, and most photonic crystal fiber designs remain at the proof-of-concept or simulation stage. They demonstrate important capabilities but require further engineering before they can be considered practical devices. This rough categorization is not meant to diminish the value of early-stage work, but to provide a clearer picture of where the field stands.

## 6. Conclusions and Future Perspectives

In conclusion, optical fiber multi-analyte biochemical sensors have demonstrated remarkable potential in the simultaneous detection of diverse biochemical targets. Through the integration of various optical transduction principles, functional nanomaterials, and biorecognition elements, these sensors have achieved high sensitivity, high specificity, and low detection limits across a broad spectrum of analytes. The ability to perform multiplexed detection in a single measurement not only improves analytical efficiency but also provides a more comprehensive understanding of complex biological and chemical systems, which is particularly valuable in precision medicine, environmental monitoring, and food safety.

Despite the challenges discussed above, the field is advancing rapidly. In the future, in-depth research on optical fiber multi-analyte biochemical sensors can be carried out in the following aspects:

1. The detection types of optical fiber multi-analyte biosensors depend on the number of modified sites and channels. In the future, the number of simultaneously detected multi-analytes can be increased by expanding the number of channels and modified sites of a single sensing probe to improve detection efficiency.
2. Optical fiber multi-analyte biochemical sensors have a high degree of miniaturized sensing probes. In the future, light sources, spectrometers, and other modules can be integrated to build an integrated detection system, increasing the compactness and portability of sensors.
3. The reliability of optical fiber multi-analyte biochemical sensors depends on their stability and repeatability in actual sample detection. In the future, more attention should be paid to the performance of sensors tested in actual samples, and the cross-sensitivity phenomenon in actual multi-analyte detection should be solved.
4. The detection method determines the detection performance of sensors to a certain extent. Exploring new signal detection methods and mechanisms is expected to improve sensor performance and broaden the detection means of optical fiber multi-analyte biochemical sensors and the types of simultaneously detected analytes.
5. Biocompatible sensors, as a pertinent research direction in biochemical sensors, are expected to realize in vivo multi-analyte detection through optical fiber multi-analyte biochemical sensors after mature technology.
6. Among the various application scenarios, liquid biopsy deserves particular attention as a high-value translational direction for optical fiber multi-analyte biosensors. Recent advances in integrated photonic sensing platforms have demonstrated the potential of lab-on-chip and lab-on-fiber technologies for highly sensitive and multiplexed biomarker detection [136]. While integrated photonic solutions such as photonic crystal waveguides and ring resonators offer high sensitivity and excellent system integration, optical fiber sensors provide complementary advantages in terms of flexibility, remote sensing capability, low-cost fabrication, and ease of deployment in point-of-care settings. Future research should focus on bridging the gap between laboratory demonstrations and clinical validation, addressing challenges such as sample matrix effects, detection reproducibility, and long-term stability in real biological fluids.

Although the current research on optical fiber multi-analyte biochemical sensors is not mature, future research can focus on these aspects to develop portable, highly integrated, low-cost, and highly reliable optical fiber multi-analyte biochemical sensors for application and popularization in daily human life.

## Figures and Tables

**Table 1:** Performance comparison of optical fiber multi-analyte biochemical sensors for the detection of multiple biomarkers and proteins.

Sensor Type	Signal	Analyte	LOD	Detection Range
SPR [43]	Intensity	Immunoglobulin G	25 μg/mL	25–200 μg/mL
		Concanavalin A		
LSPR [44]	Intensity	Streptavidin	2.92 × 10^−2^ μg/mL	0.5–50 μg/mL
		Anti-dinitrophenyl antibody	7.48 × 10^−2^ μg/mL	0.1–10 μg/mL
Fresnel Reflection [45]	Intensity	SARS-CoV-2 IgG	450 μg/mL	3.2 × 10^3^–3.508 × 10^5^ μg/mL
		SARS-CoV-2 IgM	820 μg/mL	5.2 × 10^3^–2.663 × 10^5^ μg/mL
TFBG-SPR [46]	Intensity	Aβ42 monomer	3 × 10^−6^ μg/mL	10^−4^–1 μg/mL
		Aβ42 oligomer	1.3 × 10^−5^ μg/mL	
SDI-FBG [47]	Intensity	Interleukin-6	4.8 × 10^−10^ μM	10^−11^–0.1 μM
		Interleukin-8	2.34 × 10^−8^ μM	
RAPTOR [48]	Fluorescence	Bacillus globigii	5 × 10^4^ CFU/mL	10^3^–10^6^ CFU/mL
		Ricin	0.05 μg/mL	10^−3^–1 μg/mL
		Francisella tularensis	5 × 10^5^ CFU/mL	10^4^–10^7^ CFU/mL
		Staphylococcal enterotoxin B	0.01 μg/mL	10^−4^–1 μg/mL
Tapered Fiber [49]	Fluorescence	Protein C	1.5 μg/mL	1.5–5 μg/mL
		Protein S	0.5 μg/mL	0.5–2.5 μg/mL
		Antithrombin III	45 μg/mL	45–105 μg/mL
		Plasminogen	60 μg/mL	60–120 μg/mL
		B-type natriuretic peptide	1 × 10^−4^ μg/mL	10^−4^–10^−3^ μg/mL
		Cardiac troponin I	7 × 10^−4^ μg/mL	7 × 10^−4^–7 × 10^−3^ μg/mL
		Myoglobin	0.07 μg/mL	0.07–0.7 μg/mL
		C-reactive protein	0.7 μg/mL	0.7–7 μg/mL
mPOF [50]	Fluorescence	α-Streptavidin	/	/
		Anti-C-reactive protein		
Evanescent Wave [51]	Fluorescence Chemiluminescence	miRNA-21	2.1 × 10^−7^ μM	10^−6^–10^−2^ μM
		miR-375	1.8 × 10^−8^ μM	1 × 10^−7^–5 × 10^−2^ μM
FP [52]	Phase	Anti-IgG	/	6.25–50 µg/mL
		Streptavidin		/
		Anti-laminin		
		Anti-goat IgG		
		Anti-rabbit IgG		
Microfiber [53]	Wavelength	Prostate-specific antigen	3.92 × 10^−9^ μM	10^−13^–10^−2^ μM
		Long noncoding RNA prostate cancer antigen 3	1.22 × 10^−8^ μM	

LOD and detection range values are reported as stated in the cited references and have been converted to the units shown where applicable.

**Table 2:** Performance comparison of optical fiber multi-analyte biochemical sensors for the detection of multiple viruses and bacteria.

Sensor Type	Signal	Analyte	LOD	Detection Range
Tapered Fiber [59]	Wavelength	Influenza A antigen	5.99 × 10^−5^ μg/mL	10^−6^–10^2^ μg/mL
		SARS-CoV-2 antigen	9.405 × 10^−5^ μg/mL	
Tapered Fiber [60]	Wavelength	Dengue II E protein	1 × 10^−5^ μM	10^−8^–10^−2^ μM
		SARS-CoV-2 S protein		
PCF [61]	Wavelength	*Vibrio cholerae*	1.41 × 10^−5^ RIU	/
		*Bacillus anthracis*	1.18 × 10^−5^ RIU	
		*Escherichia coli*	1.37 × 10^−5^ RIU	
		*Enterococcus faecalis*	/	
Tapered Fiber [62]	Wavelength	Hepatitis B virus	1.3 × 10^−2^ copies/mL	10^−2^–10^2^ copies/mL
		Hepatitis C virus		
Evanescent Wave [63]	Fluorescence	*Listeria monocytogenes*	1000 CFU/mL	10–10^9^ CFU/mL
		*Escherichia coli* O157:H7		
		*Salmonella enterica*		
RBFNN-POF [64]	Fluorescence	African swine fever virus (ASFV)	10^−3^ copy/mL	10^−3^–10 copies/mL
		*Salmonella*	10 CFU/mL	10–10^5^ CFU/mL

LOD and detection range values are reported as stated in the cited references and have been converted to the units shown where applicable. RIU-based values are theoretical estimates from numerical simulations and are not directly comparable to experimentally determined concentration LODs.

**Table 3:** Performance comparison of optical fiber multi-analyte biochemical sensors for the detection of multiple biological metabolites and nutrients.

Sensor Type	Signal	Analyte	LOD	Detection Range
SPR [70]	Wavelength	Vitamin K_1_	0.266 μg/mL	10^−6^–1 μg/mL
		Heparin	0.288 μg/mL	
SPR [71]	Wavelength	Glucose	0.78 μM	0–1700 μM
		Cholesterol	12 μM	0–0.3 μM
SPR [72]	Wavelength	Urea	280 μM	10^3^–9 × 10^3^ μM
		Uric acid	10.8 μM	50–500 μM
MZI-SPR-FBG [73]	Wavelength	Creatinine	70 μM	0–5 × 10^3^ μM
		Urea	90 μM	0–7 × 10^3^ μM
Tapered Fiber [74]	Fluorescence	Aflatoxin M1	21 μg/mL	1–10^6^ μg/mL
		Ochratoxin A	330 μg/mL	
Nanofiber [75]	Fluorescence	Ricin	8.6 × 10^−6^ μg/mL	5 × 10^−5^–0.14784 μg/mL
		Abrin	4 × 10^−6^ μg/mL	4 × 10^−5^–0.14025 μg/mL

LOD and detection range values are reported as stated in the cited references and have been converted to the units shown where applicable.

**Table 4:** Performance comparison of optical fiber multi-analyte biochemical sensors for the detection of multiple cells.

Sensor Type	Signal	Analyte	LOD	Detection Range
Etche-MCF-LSPR [81]	Absorbance	Human liver cancer cells HepG2	3 cells/mL	10^2^–10^6^ cells/mL
		Mouse liver cancer cells Hepa 1-6	2 cells/mL	
		Human breast cancer cells A549	2 cells/mL	
		Human lung cancer cells MCF-7	2 cells/mL	
		Human normal liver cells LO2	4 cells/mL	
		Normal canine fibroblasts NCF	10 cells/mL	
PCF-SPR [82]	Wavelength Confinement Loss	Adrenal gland cancer cells PC12	7.31 × 10^−6^ RIU	/
		Cervical cancer cells HeLa	7.31 × 10^−6^ RIU	
D-shaped PCF-SPR [83]	Wavelength Confinement Loss	Skin cancer cells Basal	2.5 × 10^−5^ RIU	/
		Cervical cancer cells HeLa	1.14 × 10^−5^ RIU	
		Blood cancer cells Jurkat	9.33 × 10^−6^ RIU	
		Adrenal gland cancer cells PC-12	6.66 × 10^−6^ RIU	
		Breast cancer cells MDA-MB-231	3.11 × 10^−6^ RIU	

LOD and detection range values are reported as stated in the cited references and have been converted to the units shown where applicable. RIU-based values are theoretical estimates from numerical simulations and are not directly comparable to experimentally determined concentration LODs.

**Table 5:** Performance comparison of optical fiber multi-analyte biochemical sensors for the detection of multiple heavy metal ions.

Sensor Type	Signal	Analyte	LOD	Detection Range
PS-LPFG [89]	Wavelength	Ni^2+^	2.014 × 10^−3^ ppm	10^−3^–10 ppm
		Zn^2+^	1.38 × 10^−3^ ppm	10^−3^–1 ppm
MMI-FRL [90]	Wavelength	Cu^2+^	0.1 μM	0.1–1 μM
		Zn^2+^		
Electrochemistry-SPR [91]	Current Wavelength	Pb^2+^	1.69 × 10^−8^ μM 5.49 × 10^−7^ μM	10^−6^–0.1 μM
		Cu^2+^	2.75 × 10^−8^ μM 5.01 × 10^−8^ μM	
MOF-SPR [92]	Wavelength	Cu^2+^	0.78 μM	0–1000 μM
		Ni^2+^	1.02 μM	
TFBG-SPR [93]	Amplitude	Pb^2+^	7 × 10^−6^ μM	10^−6^–0.1 μM
		Cd^2+^	12 × 10^−6^ μM	
		Hg^2+^	5 × 10^−6^ μM	

LOD and detection range values are reported as stated in the cited references and have been converted to the units shown where applicable.

**Table 6:** Performance comparison of optical fiber multi-analyte biochemical sensors for the detection of multiple gas.

Sensor Type	Signal	Analyte	LOD	Detection Range
MWCNT-POF [99]	Intensity	CH_3_OH	/	0–500 ppm
		C_2_H_5_OH		
Fiber CI [100]	Intensity	C_2_H_2_	20 ppm	0–2000 ppm
		NH_3_	0.01%	0–1%
LSTO-coated Fiber [101]	Intensity	H_2_	/	10–100%
		CO_2_		
		CH_4_		
		CO		
HC-ARF Raman [102]	Intensity	^13^CO_2_	0.07 ppm	/
		^12^CO_2_	0.64 ppm	
HC-ARF Raman [103]	Intensity	CH_4_	2.5 ppm	/
		C_2_H_2_	2.7 ppm	
		C_2_H_4_	2.84 ppm	
		C_2_H_6_	0.57 ppm	
		^12^CO_2_	5.13 ppm	
		^13^CO_2_	0.82 ppm	
		C_2_H_2_	/	
		CO		
		N_2_O		
Side-Polished Fiber-Planar Waveguide [104]	Wavelength	C_2_H_5_OH	1 × 10^−3^ ppm	10^−3^–10^−2^ ppm
		C_6_H_6_		
		C_2_H_7_N		
		CH_3_COOH		
		C_6_H_5_CH_3_		
FP [105]	Wavelength	CH_2_Cl_2_	4 ppm	4–400 ppm
		CHCl_3_		
		CCl_4_		
Fiber Laser-FP [106]	Amplitude	CH_4_	87 ppm	3000–10,000 ppm
		C_2_H_2_	1.3 × 10^−3^ ppm	100–1000 ppm
		CO	4.6 ppm	400–1600 ppm
		CO_2_	5.5 ppm	400–1600 ppm
		H_2_O	24 ppm	1500–5700 ppm
Fiber Ring Laser-FBG [107]	Amplitude	C_2_H_2_	0.728 ppm	200–1000 ppm
		CO_2_	503 ppm	40,000–200,000 ppm
T-type PAC [108]	Amplitude	CH_4_	6.57 × 10^−2^ ppm	250–4000 ppm
		C_2_H_2_	4.3 × 10^−3^ ppm	10–200 ppm
T-shaped Resonant Cavity-FP [109]	Amplitude	CH_4_	0.158 ppm	25–400 ppm
		C_2_H_2_	0.382 ppm	12.5–200 ppm
2 × 2 Fiber Coupler [110]	Absorbance	CO_2_	637 ppm	0–60,000 ppm
		NH_3_	0.15 ppm	0–80 ppm
Fiber Laser [111]	Absorbance	C_2_H_2_	/	400–700 ppm
		NH_3_		0.2–0.5%
Fiber Frequency Comb-PS-FBG [112]	Absorbance	CO	8.1 × 10^−6^%	2.7–8.2%
		CO_2_	/	3–15.5%
		NH_3_		0.1–0.9%
Fiber Laser [113]	Absorbance	C_2_H_2_	0.086%	1.1–2.4%
		CH_4_	0.031%	0.35–0.7%
		CO_2_	0.168%	4.5–5.4%
		H_2_O	0.0033%	0.03–0.05%
HC-ARF-FP [114]	Phase	CO	0.035 ppm	0–1000 ppm
		CO_2_	0.12 ppm	0–5000 ppm
		C_2_H_4_	0.31 ppm	0–10,000 ppm
		C_2_H_6_	0.049 ppm	0–2000 ppm
FBG-LPG-MMF [115]	Fluorescence Intensity	O_2_	/	0–6%
		CO_2_		0–5%
		NH_3_		0–2%

LOD and detection range values are reported as stated in the cited references and have been converted to the units shown where applicable.

**Table 7:** Performance comparison of optical fiber multi-analyte biochemical sensors for the detection of multiple organic pollutants.

Sensor Type	Signal	Analyte	LOD	Detection Range
Twisted POF [121]	Intensity	Methanol	/	/
		Ethanol		
		Propanol		
		Butanol		
		Pentanol		
Twisted Tapered POF [122]	Intensity	Ethanol	/	/
		Propanol		
		Butanol		
		Pentanol		
Tapered Fiber [123]	Fluorescence	Bisphenol A	6.8 μg/mL	0–10^6^ μg/mL
		2,4-dichlorophenoxyacetic acid	3.2 μg/mL	
Tapered Fiber [124]	Fluorescence	Microcystin-LR	40 μg/mL	90–1.096 × 10^5^ μg/mL
		2,4-Dichlorophenoxyacetic acid	90 μg/mL	180–9.98 × 10^4^ μg/mL
		Atrazine	20 μg/mL	40–1.105 × 10^5^ μg/mL
		Bisphenol A	30 μg/mL	50–1.101 × 10^5^ μg/mL
Evanescent Wave [125]	Fluorescence	Acetamiprid	6.51 μg/mL	14.2–225.4 μg/mL
		Fipronil	17.8 μg/mL	25.1–162.8 μg/mL
Infrared Tapered Fiber Ring [126]	Absorbance	CH_3_CH_2_OH	0.058 vol%	0.1–10 vol%
		C_6_H_5_CH_2_OH	0.1 vol%	0.3–2 vol%
		CH_3_CN	0.147 vol%	0.3–90 vol%
		C_6_H_5_CHO	0.005 vol%	0.05–0.3 vol%

LOD and detection range values are reported as stated in the cited references and have been converted to the units shown where applicable.

**Table 8:** Performance comparison of optical fiber multi-analyte biochemical sensors for the detection of different types of multiple mixed biochemical substances.

Sensor Type	Signal	Analyte	LOD	Detection Range
SPR [131]	Wavelength	DNA	5.47 × 10^−3^ μM	0–0.07 μM
		pH	0.03	5.0–9.1
PCF-SPR [132]	Wavelength Confinement Loss	SARS-CoV-2 spike RBD glycoprotein	1.953 × 10^−3^ μM	0–6.25 × 10^−2^ μM
		Ethyl alcohol	/	15–70%
HATCF-HADCF [133]	Wavelength	Glucose	40 μg/mL	0–1.2 × 10^3^ μg/mL
		pH	0.22	4.0–9.0
Optical Fiber Imaging [134]	Fluorescence	pH	/	5.5–7.5
		CO_2_		0–100%
		O_2_		0–10%
Hydrogel Fiber [135]	Fluorescence	Glucose	/	0–2 × 10^4^ μM
		pH		5.4–7.8

LOD and detection range values are reported as stated in the cited references and have been converted to the units shown where applicable.

**Table 9:** Overview of optical fiber multi-analyte biochemical sensors for different types of multiple analyte detection.

Analyte Type	Advantage	Disadvantage
Biomarkers and proteins	High sensitivity Diverse detection methods	Insufficient actual sample detection, repeatability and stability unverified
Viruses and bacteria	High sensitivity Intelligent detection	Insufficient actual sample detection, repeatability and stability unverified
Biological metabolites and nutrients	High sensitivity Low detection limit	Limited variety of analytes and insufficient actual sample detection
Heavy metal ions	High sensitivity Low detection limit High selectivity	Heavy metal ion cross-sensitivity, insufficient actual sample detection, limited types of detectable analytes
Gas	High sensitivity Low detection limit Diverse detection methods High number of simultaneously detected analytes	Limited quantitative detection and anti-environmental interference approaches
Organic pollutants	High sensitivity Good repeatability	Limited environmental interference compensation, insufficient verification for false positives and stability
Cells	High sensitivity High specificity	Insufficient verification for accuracy, repeatability, and response time
Multi-type mixed biochemical substances	Good environmental interference compensation mechanism Fast response	Insufficient real samples for detection

## Data Availability

Datasets generated during and/or analyzed during the current study are available from the corresponding author upon reasonable request.
